# A Dy(III) Coordination Polymer Material as a Dual-Functional Fluorescent Sensor for the Selective Detection of Inorganic Pollutants

**DOI:** 10.3390/molecules29184495

**Published:** 2024-09-22

**Authors:** Ying Wang, Baigang An, Si Li, Lijiang Chen, Lin Tao, Timing Fang, Lei Guan

**Affiliations:** 1 Key Laboratory of Energy Materials and Electrochemistry Research Liaoning Province, School of Chemical Engineering, University of Science and Technology Liaoning, Anshan 114051, China; ustl_wy@163.com (Y.W.);; 2 Shandong Chambroad Petrochemicals Co., Ltd., Binzhou 256500, China; 3 School of Chemistry and Chemical Engineering, Qingdao University, Qingdao 266071, China; 4 School of Petrochemical Engineering, Liaoning Petrochemical University, Fushun 113001, China

**Keywords:** coordination polymer, dysprosium, inorganic pollutant, fluorescent sensor, sensitive detection

## Abstract

A Dy(III) coordination polymer (CP), [Dy(spasds)(H_2_O)_2_]_n_ (**1**) (Na_2_Hspasds = 5-(4-sulfophenylazo)salicylic disodium salt), has been synthesized using a hydrothermal method and characterized. **1** features a 2D layered structure, where the spasda^3−^ anions act as pentadentate ligands, adopting carboxylate, sulfonate and phenolate groups to bridge with four Dy centers in *η*_3_-*μ*^1^: *μ*^2^, *η*_2_-*μ*^1^: *μ*^1^, and monodentate coordination modes, respectively. It possesses a unique (4,4)-connected net with a Schläfli symbol of {4^4^·6^2^}{4}_2_. The luminescence study revealed that **1** exhibited a broad fluorescent emission band at 392 nm. Moreover, the visual blue color has been confirmed by the CIE plot. **1** can serve as a dual-functional luminescent sensor toward Fe^3+^ and MnO_4_^−^ through the luminescence quenching effect, with limits of detection (LODs) of 9.30 × 10^−7^ and 1.19 × 10^−6^ M, respectively. The LODs are relatively low in comparison with those of the reported CP-based sensors for Fe^3+^ and MnO_4_^−^. In addition, **1** also has high selectivity and remarkable anti-interference ability, as well as good recyclability for at least five cycles. Furthermore, the potential application of the sensor for the detection of Fe^3+^ and MnO_4_^−^ was studied through simulated wastewater samples with different concentrations. The possible sensing mechanisms were investigated using Ultraviolet-Visible (UV-Vis) absorption spectroscopy and density functional theory (DFT) calculations. The results revealed that the luminescence turn-off effects toward Fe^3+^ and MnO_4_^−^ were caused by competitive absorption and photoinduced electron transfer (PET), and competitive absorption and inner filter effect (IFE), respectively.

## 1. Introduction

Coordination polymers (CPs) are an important class of functional materials, combining the merits of metal ions and organic linkers [1]. Their topological structures and physicochemical properties can be adjusted by changing organic ligands and diverse metal nodes [2]. In recent decades, CPs have attracted widespread attention due to their unique features, including their large specific surface area, tenable pore sizes and adjustable functionalities, as well as their fascinating potential applications in molecular storage, drug delivery, catalysis, ion exchange, luminescence and sensor, etc. [3,4,5,6,7]. Among these, luminescent CPs are widely used as excellent probes for the detection of environmental pollutants, including heavy metal ions, toxic inorganic anions, small molecules, pesticides, and antibiotics in aqueous media, due to their fast detection, high sensitivity and convenient operation [8,9,10,11,12]. Nevertheless, the rational design and construction of luminescent CPs are still a great challenge, which can be significantly changed by many uncertain factors, such as metal cations, organic building blocks, temperature, solvents, pH value and co-ligands, etc. [13,14,15]. From a synthetic perspective, the appropriate linkers play a crucial role in the assembly of unique topologies with excellent performances, and some aromatic ligands with N- and O-donors have been extensively employed [16,17,18]. Furthermore, lanthanide ions are preferred for the construction of luminescent CPs since they possess larger radius, diverse coordination geometries, higher affinity to negatively charged O-donor of organic ligands, and unique luminescent properties [19,20,21]. Dysprosium is considered one of the most attractive luminescent centers due to its high color purity and long lifetime arising from the 4f electrons in the excited state. Due to their unusual optical and electronic properties, Dy(III) CPs are regarded as superior fluorescence probes for sensing applications [19,20]. The research focused on luminescent Dy(III) CPs for detection have demonstrated significant sensing capabilities. However, increasing selectivity and the ability to distinguish multiple analytes remain the main focus for probes, which greatly limits the practical application of the sensing platforms.

The rapid development of society has brought a comfortable and convenient life; however, certain drawbacks like water pollution and food contamination have also been introduced to our life along with it [22]. Pollution is mainly caused by industrial wastewater discharge, in which Fe^3+^ and MnO_4_^−^ are two typical inorganic pollutants [23]. Fe^3+^ is an important element in the human body. However, if an individual is allergic to Fe^3+^, they may experience allergic reactions upon contact, such as skin itching, papules, and, in severe cases, difficulty breathing [24]. In addition, prolonged contact of skin with MnO_4_^−^ can lead to skin allergy and may increase the risk of skin cancer [25]. Owing to their potential risks to human health, there is growing worldwide concern about the development of sensitive sensors for the detection of these toxic species.

We selected 5-(4-Sulfophenylazo)salicylic disodium salt (Na_2_Hspasds) as a ligand to construct a luminescent CP, mainly based on the following three characteristics: (1) it features three functional groups containing O-donor coordination sites, which are beneficial for fabricating CPs with intriguing topological structures; (2) it possesses three different functional groups, which can play a synergistic coordination effect, leading to the formation of complicated high-dimensional structures; (3) the introduction of azobenzene groups increases the π-conjugated system and provides an excellent photosensitive performance.

In this work, we successfully synthesized a Dy(III) CP and studied its photoluminescence properties and luminescent sensing behaviors toward Fe^3+^ and MnO_4_^−^ in water via fluorescence quenching effect.

## 2. Results

### 2.1. Structure Description of ***1***

**Figure 1:**
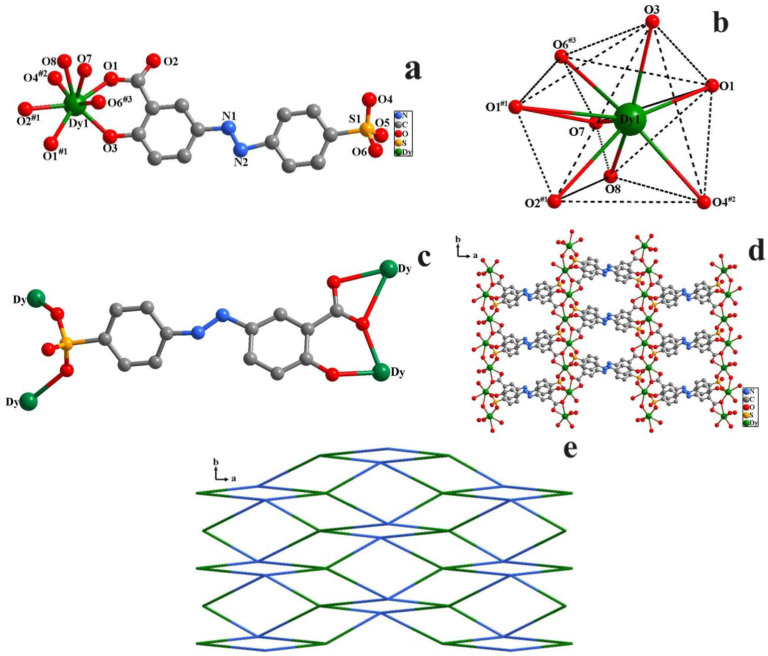
(**a**) The coordination environment of Dy^3+^ (symmetry codes #1: 2−*x*, −*y*, 4−*z*; #2: 1+*x*, 1−*y*, 2+*z*; #3: 2.5−*x*, 0.5−*y*, 5−*z*); (**b**) double-capped triangular prism coordination geometry of Dy^3+^; (**c**) coordination mode of spasds^3−^ anion; (**d**) 2D layered structure constructed by spasds^3−^ anions and Dy^3+^ cations; (**e**) (4,4)-connected topological structure.

The spasds^3−^ anions were self-assembled with Dy(NO_3_)_3_·6H_2_O to generate a 2D CP under hydrothermal condition. The single crystal X-ray analysis revealed that **1** belonged to the *C*2/*m* space group of the monoclinic crystal system (Table 1). The asymmetric unit of **1** contains one Dy(III), a deprotonated spasds^3−^ anion as organic ligand, and two coordinated water molecules (Figure 1a). Each Dy(III) center in **1** is octacoordinated with three carboxylate oxygen atoms from two spasds^3−^ ligands, one phenolate oxygen atom from one spasds^3−^ ligand, two sulfonate oxygen atoms from two spasds^3−^ linkers, and two oxygen atoms in two coordinated water molecules, which gives it a double-capped triangular prism coordination geometry (Figure 1b). The bond lengths of Dy–O are in the range of 2.2191(240)–2.6973(266) Å (Table S1); the bond angles of O–Dy–O are between 50.382(647) and 154.447(828)° (Table S1). All the bond lengths and angles around the Dy(III) centers are within the range of the values previously reported for other Dy CPs [26,27]. One carboxylate group of the spasds^3−^ anion coordinates to two Dy(III) ions in a *η*_3_-*μ*^1^: *μ*^2^ coordination mode, the sulfonate group bridges with two Dy(III) ions in a *η*_2_-*μ*^1^: *μ*^1^ coordination fashion, and the phenolate group binds to one Dy(III) ion in a monodentate mode (Figure 1c). In **1**, spasds^3−^ anions utilize the sulfonate, carboxylate and phenolate groups to link with four adjacent Dy(III) ions, generating a 2D layered structure (Figure 1d). Topologically, each Dy(III) center is linked by four neighboring spasds^3−^ ligands, which can act as a 4-connected node, and each spasds^3−^ ligand connects to four Dy(III) centers, which can be described as a 4-connected spasds^3−^ ligand-based node. With further topological analysis using ToposPro software (Version 5.5.2.2) [28], the framework of **1** can be simplified to a unusual (4,4)-connected topological structure with the point symbol of {4^4^·6^2^}{4}_2_ (Figure 1e).

### 2.2. Thermogravimetric Analysis

The thermal stability of **1** was studied by thermogravimetric analysis (TGA) experiment under a nitrogen atmosphere in the temperature range of 25–800 °C (Figure S1). The TGA curve of **1** displays a weight loss of 7.8% from 40 to 118 °C, agreeing to the loss of two coordinated water molecules (calcd 7.0%). As the temperature increases, the spasds^3−^ ligand gradually undergoes thermal decomposition. After 460 °C, the rapid weight loss may be caused by the breakdown of the spasds^3−^ ligand. In addition, the weight loss slows after 545 °C, and the decomposition is still not complete when the temperature reaches 800 °C.

### 2.3. Powder X-ray Diffraction

In order to check the phase purity of the synthesized product, the powder X-ray diffraction (PXRD) pattern was conducted on the solid powder sample of **1** (Figure S2). The measured PXRD pattern was consistent with those simulated based on the single crystal data, indicating the good phase purity of **1**. In addition, the PXRD pattern of sample of **1**, after soaking in deionized water for 7 days, remained unchanged, manifesting that **1** kept its crystallinity and had high stability in water.

### 2.4. Scanning Electron Microscopy

The morphology of the sample of **1** was observed using a scanning electron microscope (SEM) and shown in Figure S3. SEM confirms that the particles of sample **1** exist individually and have a regular morphology, with a particle size of approximately 200–1000 nm.

### 2.5. Photoluminescence Properties

**Figure 2:**
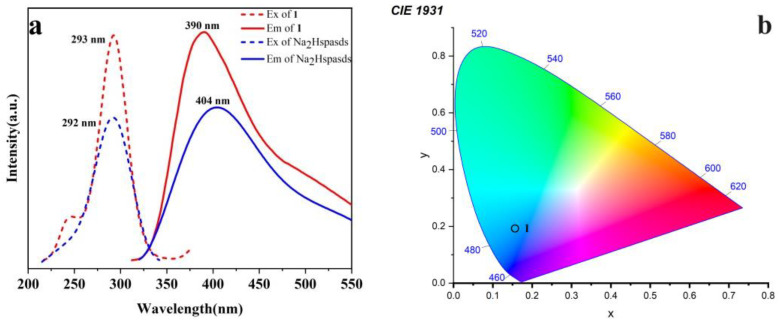
Fluorescence excitation and emission spectra of (**a**) free Na_2_Hspasda ligand and **1**; (**b**) CIE plot of **1** (**1** refers to the title CP. The circle indicates the color).

Ligand fluorescence is an attractive research topic for selective detection. Therefore, the solid-state luminescence spectra of the Na_2_Hspasds ligand and **1** were recorded at room temperature (Figure 2a). The ligand exhibits a wide emission band at 404 nm under excitation at 362 nm, which originates from π → π* and/or n → π* transitions within the ligand. Likewise, a similar broad emission band for **1**, centered at 390 nm, was observed when excited at 292 nm, which can be assigned to a ligand-centered transition [29]. Generally, the antenna effect can sensitize the central rare earth metal ion and make it exhibit the characteristic fluorescence emission. However, the azo and sulfonate groups in the Na_2_Hspasds ligand are strong electron acceptors, which can passivate the antenna effect [30]. Therefore, the Dy(III) ions in **1** cannot exhibit the characteristic fluorescence emission, but instead display the fluorescence emission of the ligand.

The emission band of **1** shows a blue shift (Δ = 14 nm) with respect to the free Na_2_Hspasds. The shift of the emission band in **1** relative to that of the Na_2_Hspasds ligand may be caused by ligand-to-metal charge transition [31]. Calculation of the CIE chrominance coordinate for **1** is shown in Figure 2b, the CIE coordinate of **1** is located in the blue-violet region at (0.1568, 0.1935).

### 2.6. Selective Sensing of Fe^3+^

CPs constructed from *d*^10^ metal ions/lanthanide metal ions and organic ligands, are often used as fluorescent sensors to detect pollutants in the environment. Thus, **1** is a potential candidate for luminescent probes, and its properties encourage us to systematically investigate its detection performance.

**Figure 3:**
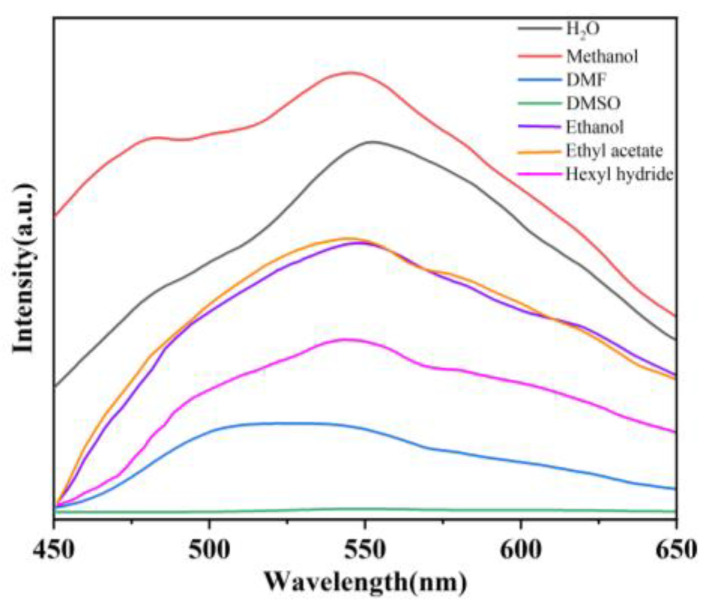
Fluorescence responses of **1** toward different solvents (*λ*_ex_ = 370 nm).

Seven common solvents [water, methanol, *N*, *N*-dimethylformamide (DMF), dimethyl sulfoxide (DMSO), ethanol, ethyl acetate and hexyl hydride] were selected to investigate the fluorescence emission of the suspension of **1** (Figure 3). 3 mg of ground powder samples of **1** were immersed in 3 mL of different solvents, ultrasonicated for 15 min, and then left to stand for 2 h to form stable suspensions for fluorescent analysis. **1** showed the strongest emission in the suspensions of methanol and water. Considering that methanol is an organic solvent that may negatively impact water bodies and soil, while water is more environmentally friendly, we used water as the dispersion medium in the following sensing experiments.

**Figure 4:**
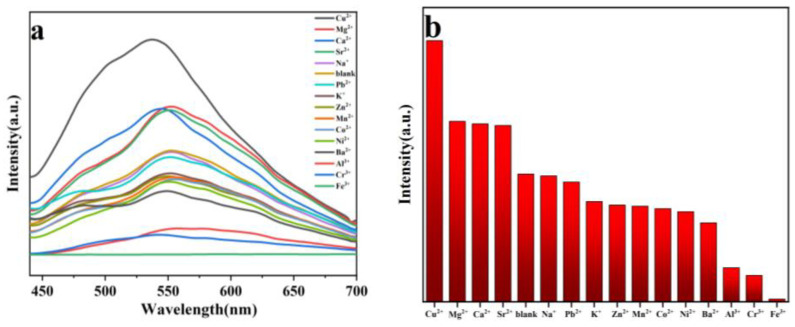
(**a**) Fluorescence responses; and (**b**) fluorescence intensities of **1** toward different metal cations at 0.01 M in aqueous solution (*λ*_ex_ = 370 nm).

To investigate the fluorescence sensing behaviors of **1** toward different metal ions, each of the aqueous solutions of metal chlorides (MCl_n_, M^n+^ = Cu^2+^, Mg^2+^, Ca^2+^, Sr^2+^, Na^+^, Pb^2+^, K^+^, Zn^2+^, Mn^2+^, Co^2+^, Ni^2+^, Ba^2+^, Al^3+^, Cr^3+^, Fe^3+^) was added to the aqueous suspension of **1**, then the fluorescence spectra of the mixed aqueous suspensions were recorded when excited at 370 nm (Figure 4a,b). We found that the luminescence intensities of the aqueous suspensions of **1** are significantly dependent on the type of metal ions. Interestingly, **1** displays the most significant quenching behavior with a quenching percentage ((1 − I/I_0_) × 100%) of 98% for Fe^3+^. Our experimental observations indicate that **1** has specific recognition of Fe^3+^ in aqueous medium.

**Figure 5:**
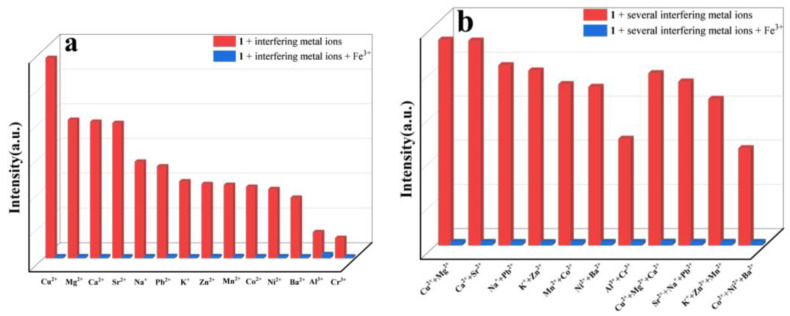
Competitive experiments of the suspensions of **1** with (**a**) one interfering metal ion, and (**b**) several interfering metal ions (*λ*_ex_ = 370 nm).

To further explore the effect of different interfering ions on detecting Fe^3+^, competitive experiments were carried out (Figure 5a,b). When Fe^3+^ was added to the aqueous suspension of **1** with one interfering metal ion (Cu^2+^, Mg^2+^, Ca^2+^, Sr^2+^, Na^+^, Pb^2+^, K^+^, Zn^2+^, Mn^2+^, Co^2+^, Ni^2+^, Ba^2+^, Al^3+^ or Cr^3+^), the quenching phenomenon was similar to that without interfering metal ions. Moreover, when two or three interfering metal ions were present, the fluorescence quenching effect of Fe^3+^ on the suspension of **1** was not affected. These results reveal that **1** could serve as a promising luminescent probe for the highly selective detection of Fe^3+^, which is not affected in the presence of interfering metal ions.

**Figure 6:**
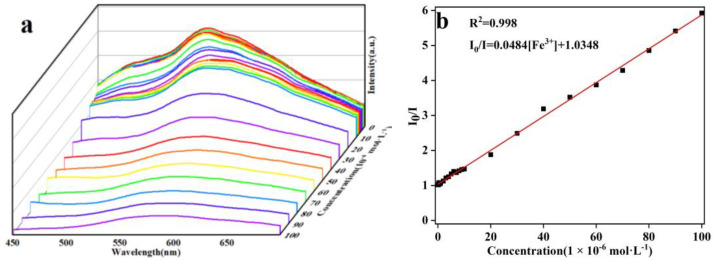
(**a**) Fluorescence responses of **1** with the dropwise addition of the aqueous solution of Fe^3+^; (**b**) Stern–Volmer plot for the quenching effect of Fe^3+^ on **1** (*λ*_ex_ = 370 nm). (The different color lines and square shapes represent different concentrations).

To examine the sensitivity toward Fe^3+^, Fe^3+^ was added dropwise to the aqueous suspension of **1** for fluorescence-quenching titration. The fluorescence intensity of **1** gradually decreased as the concentration of Fe^3+^ gradually increased (Figure 6a). The relationship between quenching efficiency and the concentration of Fe^3+^ was analyzed using the Stern–Volmer equation: (I_0_/I) = K_SV_[M] + 1 (I_0_ for the initial luminescence intensity, I for the luminescence intensity after the addition of the solution of Fe^3+^, [M] for the molar concentration of Fe^3+^ and K_SV_ for the Stern–Volmer constant). A good linear correlation is obtained between Fe^3+^ concentration and I_0_/I, with a correlation coefficient (R^2^) of 0.998 and a K_SV_ value of 4.84 × 10^4^ M^−1^ (Figure 6b). The LOD was calculated to be 9.30 × 10^−7^ M using the equation of LOD = 3σ/K_sv_, where σ represents the standard deviation from ten instrumental blank measurements [32]. The LOD is relatively low in comparison with those of the reported CP-based sensors for Fe^3+^ (Table 2) [33,34,35,36,37,38,39,40,41]. The results demonstrate that **1** shows an efficient detection sensitivity toward Fe^3+^ in a water medium.

**Figure 7:**
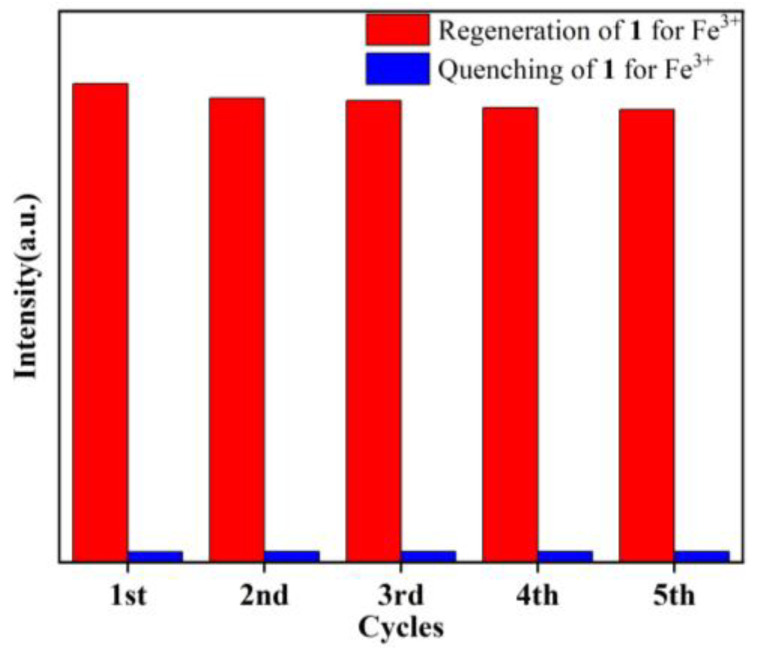
Quenching and regeneration experiments of **1** for the detection of Fe^3+^.

To further test the recyclable performance of the **1**-based chemical sensor in detecting Fe^3+^, its reproducibility was evaluated through reuse experiments (Figure 7). After each sensing experiment, the suspensions of **1** were centrifuged and filtered, and then washed with deionized water several times. The crystal sample of **1** was easily recollected and regenerated. The luminescent intensities of **1** did not change significantly and the suspension of **1** still maintained high selectivity for sensing Fe^3+^ after five cycles. The experimental results prove the recyclability of **1** for the detection of Fe^3+^.

### 2.7. Selective Sensing of MnO_4_^−^

Permanganate (MnO_4_^−^) salts, a class of strong oxidants, have been widely used in chemical production, organic synthesis, as bactericides, and in bleaching. However, exposure to MnO_4_^−^, even in fairly small amounts, can corrode the skin and irritate human tissues. Excessive MnO_4_^−^, as an environmental pollutant, can disrupt microbial communities and affect the ecological balance in the soil, especially, causing plant wilting and death. Thus, it is essential to directly and effectively detect trace amounts of MnO_4_^−^.

**Figure 8:**
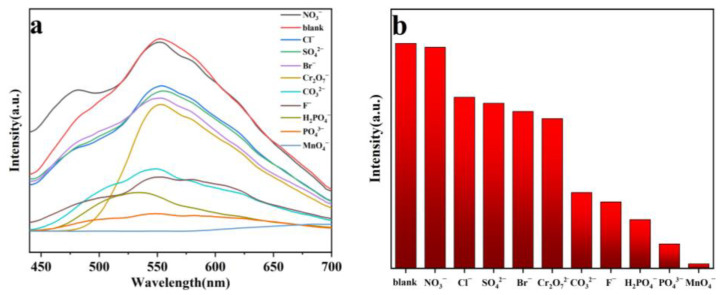
(**a**) Fluorescence responses; and (**b**) fluorescence intensities of **1** toward different inorganic anions at 0.01 M in aqueous solution (*λ*_ex_ = 370 nm).

**Figure 9:**
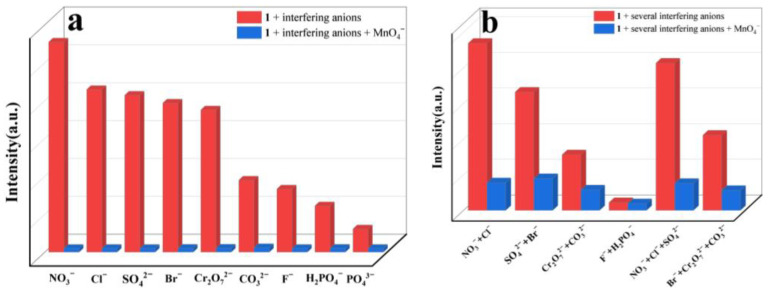
Competitive experiments of the suspensions of **1** with (**a**) one interfering anion; and (**b**) several interfering anions (*λ*_ex_ = 370 nm).

**Figure 10:**
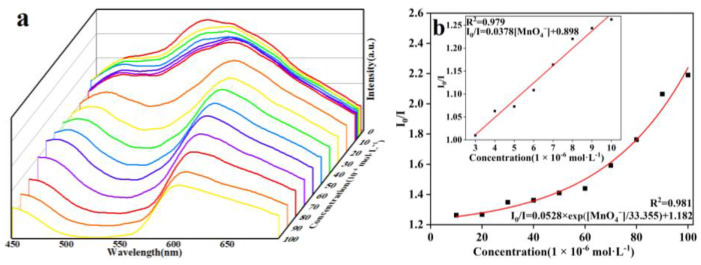
(**a**) Fluorescence responses of **1** with the dropwise addition of the aqueous solution of MnO_4_^−^; (**b**) Stern–Volmer plot for the quenching effect of MnO_4_^−^ on **1** (*λ*_ex_ = 370 nm). (The different color lines and square shapes represent different concentrations).

**Figure 11:**
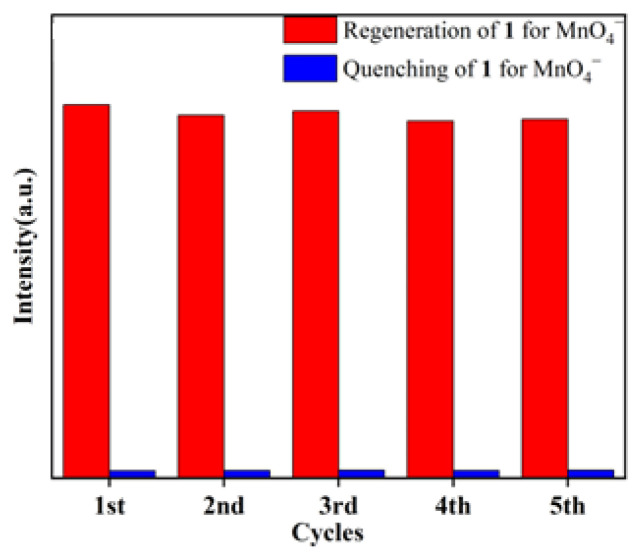
Quenching and regeneration experiments of **1** for the detection of MnO_4_^−^.

The same process used for Fe^3+^ sensing was applied to investigate its sensing performance toward different inorganic anions (Figure 8a). The ground sample of **1** was dispersed in various aqueous solutions of potassium salt, K_n_X (NO_3_^−^, Cl^−^, SO_4_^2−^, Br^−^, Cr_2_O_7_^2−^, CO_3_^2−^, F^−^, H_2_PO_4_^−^, PO_4_^3−^ and MnO_4_^−^). It was observed that the anions caused different extents of luminescent turn-off effects; only MnO_4_^−^ could efficiently cause significant fluorescence quenching in the suspension of **1**, with a quenching efficiency of 98% (Figure 8b), revealing that MnO_4_^−^ could be detected by **1**. To evaluate the selectivity of **1**, competitive experiments were performed (Figure 9a,b). No significant changes in emission intensity were found in the presence of one interfering anion, and several interfering anions, indicating that **1** has good anti-interference ability for detecting MnO_4_^−^. To further evaluate the ability of **1** for sensing a trace amount of MnO_4_^−^, titration experiments of fluorescence quenching were carried out. The luminescent intensities of the suspension of **1** sequentially decreased as MnO_4_^−^ solution was added dropwise to the suspension of **1** (Figure 10a). The quenching effects of the MnO_4_^−^ can be fitted by the exponential quenching formula: I_0_/I = 0.0528 × exp([MnO_4_^−^]/33.355) + 1.182, where I_0_ and I are the luminescence intensities before and after the addition of the solution of MnO_4_^−^, respectively, and [MnO_4_^−^] is the molar concentration of MnO_4_^−^. When MnO_4_^−^ is at a low concentration, the linear relationship between the concentration and the luminescence intensities can be fitted with the Stern–Volmer equation of I_0_/I = 0.0378[MnO_4_^−^] + 0.898 (Figure 10b). A good linear correlation (R^2^) of 0.979 was observed and the K_sv_ value was 3.78 × 10^4^ M^−1^. The LOD was calculated to be 1.19 × 10^−6^ M with the equation of 3σ/K_sv_, indicating a lower detection limit than some previously reported CP-based luminescent sensors for the detection of MnO_4_^−^ (Table 3) [42,43,44,45,46,47,48,49]. In addition, the reusability of the fluorescence sensor is very important for **1** to detect MnO_4_^−^. After each detection, the sample of **1** was easily recollected and reproduced by centrifuging and washing it multiple times with deionized water. After five cycles, the sample of **1** can still maintain a strong fluorescence emission and good quenching efficiency for MnO_4_^−^ (Figure 11).

### 2.8. Practical Application

**Figure 12:**
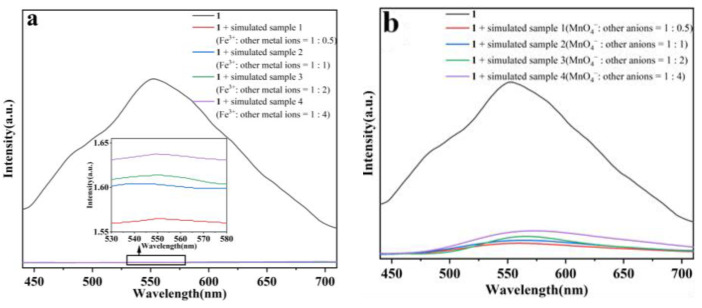
(**a**,**b**) Comparison of luminescence changes before and after the addition of the simulated samples.

To further investigate the feasibility of the designed Dy(III) CP sensor for Fe^3+^ and MnO_4_^−^ in actual samples, four types of artificial wastewater were selected as real samples for analysis, which were prepared with different concentrations of interfering ions. A certain amount of Fe^3+^/MnO_4_^−^ was added to the simulated solution at different ratios to the interfering ions, and then the mixed solutions were added to the suspensions of **1** after ultrasonic uniformity. The luminescence of each concentration was measured at room temperature, and the luminescence changes before and after the addition of the simulated samples were compared. The luminescence intensities significantly changed, as can be seen in Figure 12a,b, which proved that **1** has good practicality.

### 2.9. Quenching Mechanisms

**Figure 13:**
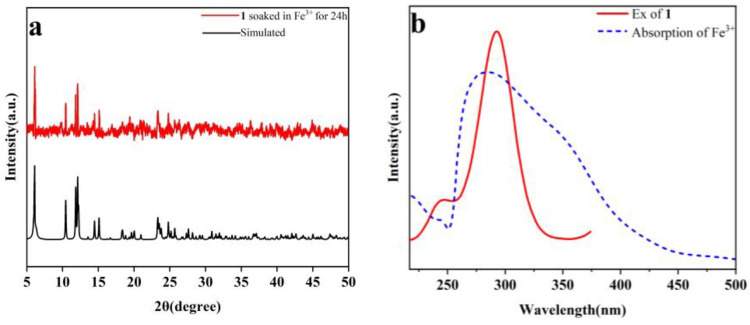
(**a**) Measured PXRD pattern of the sample of **1** after soaking in Fe^3+^ for 24 h and simulated one based on single crystal data of **1**; (**b**) UV-Vis absorption spectrum of Fe^3+^ and fluorescence excitation spectrum of **1**.

**Figure 14:**
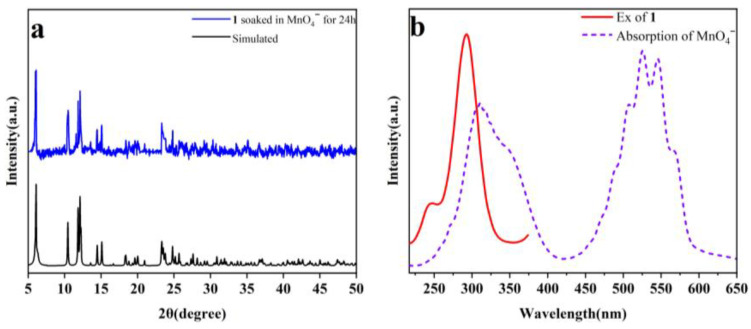
(**a**) Measured PXRD pattern of the sample of **1** after soaking in MnO_4_^−^ for 24 h and simulated one based on single crystal data of **1**; (**b**) UV-Vis absorption spectrum of MnO_4_^−^ and fluorescence excitation spectrum of **1**.

In order to study the possible quenching mechanisms of **1** toward Fe^3+^ and MnO_4_^−^, some experiments were performed. Firstly, the PXRD patterns of the samples of **1** immersed in Fe^3+^ and MnO_4_^−^ solutions are in good agreement with the simulated one (Figure 13a and Figure 14a). The results revealed that the structure of **1** can remain intact after treatment with Fe^3+^ and MnO_4_^−^, indicating that the quenching effect cannot be attributed to the collapse of the framework. Secondly, the exchange of the central ions (Dy^3+^) with Fe^3+^ is very difficult and takes some time, but the quenching phenomenon quickly occurs when the solution of Fe^3+^ is added to the suspension of **1**. Thirdly, according to the UV-Vis absorption spectra, Fe^3+^ and MnO_4_^−^ exhibit clear overlaps with the excitation band of **1** (Figure 13b and Figure 14b). Thus, competitive absorption may be responsible for the selective quenching effects [50]. Finally, to verify if there are interactions between the analytes and **1**, X-ray photoelectron spectroscopy (XPS) experiments were carried out on the sample of **1** before and after treatment with aqueous solutions of Fe^3+^ and MnO_4_^−^ (Figure S4). The results of XPS indicated no obvious peak shifts, and new peaks were found on the samples of **1** after treatment with Fe^3+^ and MnO_4_^−^, in contrast to the original sample, revealing that there are no weak interactions between the analytes and **1**. Furthermore, their IR spectra can also confirm this result (Figure S5).

**Figure 15:**
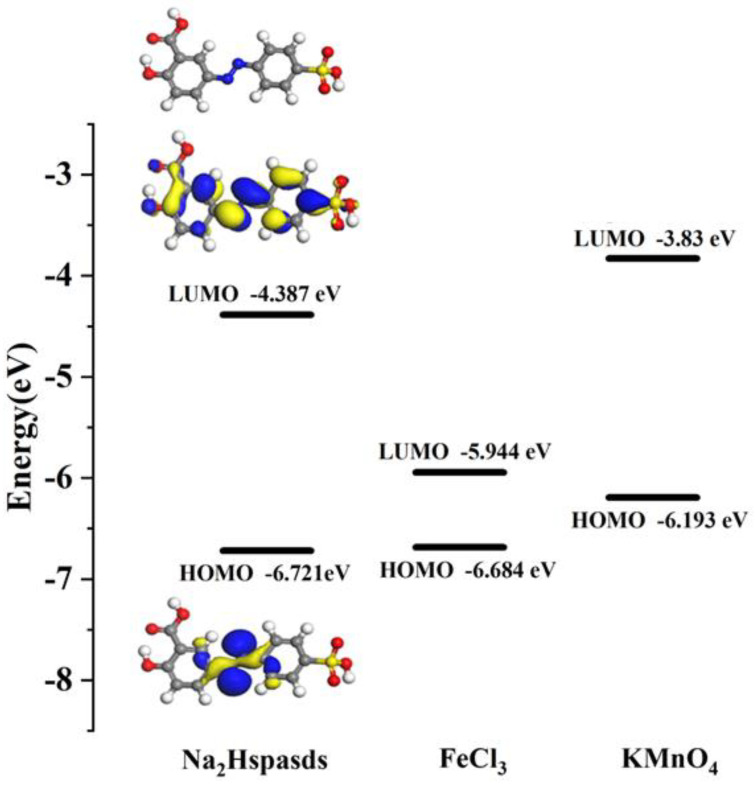
Structure optimization and the frontier molecular orbital energy levels of the ligand, Fe^3+^ and MnO_4_^−^.

To further explore the mechanisms of fluorescence turn-off effects, we carried out geometry optimizations and theoretical calculations of their frontier molecular orbital energy levels using the DMol^3^ package in the Material Studio 2018 software. According to the DFT, the HOMO and LUMO energy levels of the ligand, Fe^3+^ and MnO_4_^−^ were calculated (Figure 15). It was observed that the LUMO level of the ligand possesses higher energy than the LUMO energy level of Fe^3+^. Therefore, it makes the charge transfer much easier from the excited ligand to the LUMO of Fe^3+^, causing the significant fluorescence turn-off effect. The results indicate that the photo-induced electron transfer (PET) process is also pivotal in detecting Fe^3+^ [50]. However, the relatively higher LUMO level of MnO_4_^−^ with respect to the LUMO of the ligand ruled out the charge transfer from the ligand to MnO_4_^−^, indicating the absence of the PET process, and the relatively higher HOMO level of MnO_4_^−^ compared to the HOMO of the ligand can confirm that the inner filter effect (IFE) also plays an influential role in the fluorescence quenching toward MnO_4_^−^, which usually occurs when the excitation spectrum of CP overlaps with the absorption spectrum of the analyte [51]. Based on the above studies, we could speculate that the fluorescence quenching toward Fe^3+^ and MnO_4_^−^ can be ascribed to the competitive adsorption and PET effect, and competitive adsorption and IFE effect, respectively.

## 3. Experimental Section

### 3.1. Materials and Methods

All chemical reagents and different solvents were purchased from Shanghai Macklin Biochemical Technology Co., Ltd., Shanghai City, China. They were of reagent grade and directly used without further treatment. Elemental analysis was performed using a Perkin-Elmer 240CHN analyzer (Perkin-Elmer Corporation, Norwalk, CT, USA). FT-IR spectra were recorded as KBr pellets using an Alpha spectrometer (Bruker, Rheinstetten, Germany) between 4000 cm^−1^ and 400 cm^−1^. Thermogravimetric analysis was performed using an SDT-Q600 (TA Corporation, New Castle, DE, USA) thermogravimetric analyzer between 25 and 800 °C (nitrogen atmosphere, heating rate of 10 K·min^−1^), with an Al_2_O_3_ crucible. After heat treatment and drying to remove adsorbed and free water molecules, the sample was placed in an Al_2_O_3_ crucible. Powder X-ray diffraction (PXRD) patterns were collected using a D/MAX-2500X XRD diffractometer (Rigaku X’pret, Tokyo, Japan) with a Cu-K_α_ monochromator (Cu-K_α_ radiation, λ = 1.5406 Å). The calculated PXRD pattern was simulated using the single crystal X-ray diffraction data. The morphology of the sample was characterized using an Apreo field-emission scanning electron microscope (FEI Corporation, Hillsboro, OR, USA). Fluorescence excitation and emission spectra were measured using a Perkin-Elmer LS55 fluorescence spectrophotometer (Perkin-Elmer Corporation, Norwalk, CT, USA), equipped with a 75W Xenon arc lamp and an R928 photomultiplier tube (Perkin-Elmer Corporation, Norwalk, CT, USA) as a detector for solid samples. Emission intensity measurements were carried out using the adapter and holder supplied by the manufacturer of the spectrophotometer. Excitation and emission spectra were corrected for the instrumental response. UV-Vis absorption spectra were measured using a Cary5000 spectrophotometer (Agilent Corporation, Santa Clara, CA, USA). X-ray photoelectron spectroscopy was performed using an AXIS-SUPRA spectrometer (SHIMADZU Corporation, Kyoto, Japan).

### 3.2. Synthesis of ***1***

A mixture of Na_2_Hspasds (0.037 g, 0.1 mmol) and Dy(NO_3_)_3_·6H_2_O (0.046 g, 0.1 mmol) was dissolved in 10 mL of deionized water. The mixture was sealed in a Teflon-lined stainless steel vessel (15 mL) and heated at 120 °C under autogenous pressure for 2 days, and then cooled to room temperature. Yellow block-shaped crystals were obtained, which were then filtered, washed with deionized water and dried at room temperature. Yield: 67% (based on Dy). Elemental analysis (%) found: C 30.16, H 2.20, N 5.43%. Calcd for C_13_H_11_N_2_O_8_SDy: C 30.15, H 2.14, N 5.41%. IR (KBr, cm^−1^): 3079, 1601, 1552, 1514, 1481, 1456, 1422, 1342, 1218, 1165, 1120, 1030, 1003, 838, 799, 677, 612, 575 (Figure S5).

### 3.3. X-ray Crystallography

The crystallographic data for **1** were collected at 193 K using a Bruker APEX-II diffractometer equipped with a CPAD area detector. The measurements were made by using graphite monochromatic Ga-K_α_ radiation (λ = 1.34139 Å). Data reduction was performed using the SAINT program, and absorption correction was carried out using TWINABS-2012. For component 1, *wR*_2_ was 0.1167 before and 0.0773 after correction. For component 2, *wR*_2_ was 0.1208 before and 0.0800 after correction. The structure of **1** was solved by direct methods using the SHELXS program and refined by the full-matrix least-squares method on *F*^2^ using the SHELXL program [52,53]. All non-hydrogen atoms were anisotropically refined, and hydrogen atoms of the organic linker were generated theoretically on the specific atoms. The sample of **1** is twinned and the structure is disordered, which can cause the B-level alert “Large Hirshfeld Difference”. For clarity, the disordered structure was removed in the drawing. Detailed crystal data and structure refinement parameters of **1** are summarized in Table 1. The bond lengths and bond angles are given in Table S1.

### 3.4. Luminescence Sensing Experiments

All fluorescence sensing experiments were carried out by adding the powder sample of **1** (3 mg) to the solution (3 mL) of different analytes. Before the fluorescence spectrum was recorded, the mixed solution was sonicated for 15 min to form a stable suspension, which was subsequently transferred to a four-way quartz cell with 1 cm width. For the blank sample in liquid fluorescence testing, the optimal excitation wavelength for fluorescence emission was found to be 370 nm. Thus, for selectivity, anti-interference, and quantitative titration experiments, the luminescence measurements were performed using an excitation wavelength of 370 nm at room temperature.

### 3.5. Computational Approach

The geometry of the ligand was first sketched and optimized using Material Studio 2018 software. The corresponding optimized geometrical model of the ligand was further used to calculate the frontier molecular orbital energy levels based on DFT, as implemented in the DMol^3^ code. The parameters used in the calculations and the convergence criteria were based on the basis set of the DMol^3^ program in Materials Studio [54,55,56,57,58].

## 4. Conclusions

In summary, a new Dy(III) CP has been constructed using the spasds^3−^ ligand, which displays a 2D 4, 4-c {4^4^·6^2^}{4}_2_ net. It exhibits strong ligand-based fluorescence emission in the solid state. It acts as a dual-responsive luminescent sensor for Fe^3+^ and MnO_4_^−^ in aqueous solution through fluorescence quenching effects. **1** also exhibits several remarkable features including high selectivity, anti-interference, sensitivity, excellent recyclability, and potential practicality. The LODs are 9.30 × 10^−7^ and 1.19 × 10^−6^ M, respectively. Moreover, the possible mechanisms of luminescence sensing toward Fe^3+^ and MnO_4_^−^ are ascribed to the combination of competitive adsorption and PET, and the combination of competitive adsorption and IFE, respectively.

## Figures and Tables

**Table 1:** Detailed crystal data and structure refinement parameters of **1**.

	1
Chemical formula	C_13_H_11_N_2_O_8_SDy
*M*_r_	517.8
Crystal system	Monoclinic
Space group	*C*2/*m*
Temperature (K)	193
*a*, *b*, *c* (Å)	29.6268(18), 8.8632(6), 7.6514(5)
β (°)	102.436(4)
*V* (Å^3^)	1962.0(2)
*Z*	4
*V* (Å^3^)	1962.0(2)
*µ* (mm^−1^)	20.6
*T*_min_, *T*_max_	0.442, 0.751
No. of measured, independent and	1862, 1862, 1724
observed [*I* > 2*σ*(*I*)] reflections	
*R*_int_	0.0903
(sin*θ*/*λ*)_max_ (Å^−1^)	0.602
*R*[*F*^2^ > 2σ(*F*^2^)], *wR*(*F*^2^), *S*	0.085, 0.219, 1.12
No. of reflections	1862
No. of parameters	201
No. of restraints	188
*R*_1_, *ωR*_2_ [*I* ≥ 2*σ*(*I*)]	0.0848, 0.2074
*R*_1_, *ωR*_2_ (all data)	0.0966, 0.2185
Δ*ρ*_max_, Δ*ρ*_min_ (e·Å^−3^)	2.79, −2.25

**Table 2:** Comparison of LODs between **1** and the reported CP-based sensors for Fe^3+^.

Sensor	K_sv_/M^−1^	LOD/M	Reference
[Dy(spasds)(H_2_O)_2_]_n_	4.84 × 10^4^	9.30 × 10^−7^	This work
[Co(L)(TBTA)(H_2_O)_2_·H_2_O]_n_	1.42 × 10^4^	1.61 × 10^−6^	[33]
[Zn(L)_2_·2DMF]_n_	1.92 × 10^4^	2.34 × 10^−5^	[34]
[Cd(bimbp)(3-HNA)_2_]_n_	4.39 × 10^4^	5.9 × 10^−5^	[35]
[Zn(L)·H_2_O]_n_	1.38 × 10^4^	3.01 × 10^−5^	[36]
[Cd(L)(nip)(H_2_O)·H_2_O]_n_	5.53 × 10^3^	7.16 × 10^−6^	[37]
[Cd(btic)(phen)·0.5H_2_O]_n_	4.959 × 10^4^	1.524 × 10^−4^	[38]
[Zn(Hssa)(1,4-bib)·H_2_O]_n_	4.16 × 10^4^	1.66 × 10^−6^	[39]
[Eu(BDC)_1.5_(IP)(H_2_O)]·nH_2_O	2.33 × 10^4^	6.05 × 10^−5^	[40]
[Tb_2_(ccpc)_2_(H_2_O)_6_]·1.5H_2_O	1.022 × 10^4^	1.71 × 10^−6^	[41]

**Table 3:** Comparison of LODs between **1** and the reported CP-based sensors for MnO_4_^−^.

Sensor	K_sv_/M^−1^	LOD/M	Reference
[Dy(spasds)(H_2_O)_2_]_n_	3.78 × 10^4^	1.19 × 10^−6^	This work
[Co(NPDC)(bpee)·DMF·2H_2_O]_n_	4.26 × 10^3^	1.5 × 10^−6^	[42]
[Co_2_(L2)(1,4-chdc)_2_]_n_	1.63 × 10^4^	1.2 × 10^−4^	[43]
[Zn(L)_2_·2DMF]_n_	1.92 × 10^4^	2.34 × 10^−5^	[34]
[Zn(BTC)(Hdpa)·H_2_O]_n_	2.46 × 10^4^	8.86 × 10^−6^	[44]
[Cd(L)_2_(H_2_O)_2_]_n_	2.2 × 10^4^	1.73 × 10^−4^	[45]
[Zn(3-bpat)(5-mip)]_n_	2.8 × 10^4^	3.64 × 10^−6^	[46]
[Zn_2_(OH)_2_(1,5-NDS)(Mbimb)]_n_	7.3 × 10^3^	4.33 × 10^−6^	[47]
[Eu_2_Na(Hpddb)(pddb)_2_(CH_3_COO)_2_·2.5(DMA)]_n_	2.84 × 10^3^	5.99 × 10^−6^	[48]
[Tb(H_2_O)(L)(TPA)·0.5H_2_O]_n_	1.13 × 10^4^	1.43 × 10^−6^	[49]

## Data Availability

The data presented in this study are available on request from the corresponding author.

## Supplementary Materials

The following supporting information can be downloaded at: https://www.mdpi.com/article/10.3390/molecules29184495/s1, Figure S1: TGA curve of **1**; Figure S2: Measured PXRD patterns of the samples of **1** before and after soaking in water for 7 d, and simulated one based on single crystal data of **1**; Figure S3: SEM profile of **1**; Figure S4: XPS spectra of the samples of **1** before and after treatment with Fe^3+^ and MnO_4_^−^; Figure S5: IR spectra of the samples of **1** before and after treatment with Fe^3+^ and MnO_4_^−^; Table S1: Selected bond lengths (Å) and angles (°) for **1**.
