# Supplementary material for: A Dy(III) Coordination Polymer Material as a Dual-Functional Fluorescent Sensor for the Selective Detection of Inorganic Pollutants
Source: Molecules. 2024 Sep 22;29(18):4495. doi: 10.3390/molecules29184495 (PMC11435080; doi:10.3390/molecules29184495)

checkCIF/PLATON report

You have not supplied any structure factors. As a result the full set of tests cannot be run.

THIS REPORT IS FOR GUIDANCE ONLY. IF USED AS PART OF A REVIEW PROCEDURE FOR PUBLICATION, IT SHOULD NOT REPLACE THE EXPERTISE OF AN EXPERIENCED CRYSTALLOGRAPHIC REFEREE.

No syntax errors found. CIF dictionary Interpreting this report

Datablock: 1

Bond precision:	= 0.0000 A		Wavelength=1.34139
Cell:	a=29.6268(18)	b=8.8632(6)	c=7.6514(5)
	alpha=90	beta=102.436(4)	gamma=90
Temperature:	193 K		

	Calculated	Reported
Volume	1962.0(2)	1962.0(2)
Space group	C 2/m	C 1 2/m 1
Hall group	-C 2y	-C 2y
Moiety formula	C52 H44 Dy4 N8 O32 S4 [+ solvent]	C13 H11 Dy N2 O8 S
Sum formula	C52 H44 Dy4 N8 O32 S4 [+ solvent]	C13 H11 Dy N2 O8 S
Mr	2071.19	517.80
Dx,g cm-3	1.753	1.753
Z	1	4
Mu (mm-1)	20.601	20.601
F000	996.0	996.0
F000’	976.52	
h,k,lmax	35,10,9	35,10,9
Nref	1925	1862
Tmin,Tmax	0.127,0.157	0.442,0.751
Tmin’	0.056	

Correction method= # Reported T Limits: Tmin=0.442 Tmax=0.751
AbsCorr = MULTI-SCAN

Data completeness= 0.967 Theta(max)= 53.893

```
R(reflections)= 0.0848( 1724)          wR2(reflections)= 0.2185( 1862)
S = 1.115                 Npar= 201
```

The following ALERTS were generated. Each ALERT has the format
test-name_ALERT_alert-type_alert-level.
Click on the hyperlinks for more details of the test.

Alert level B

```
PLAT234_ALERT_4_B Large Hirshfeld Difference Dy1      --O7       .       0.26 Ang.
```

Alert level C

```
PLAT088_ALERT_3_C Poor Data / Parameter Ratio ....................       9.58 Note
PLAT234_ALERT_4_C Large Hirshfeld Difference Dy1      --O7A      .       0.21 Ang.
PLAT242_ALERT_2_C Low    'MainMol' Ueq as Compared to Neighbors of        Dy1 Check
```

Alert level G

```
ABSMU01_ALERT_1_G  Calculation of _exptl_absorpt_correction_mu
                not performed for this radiation type.
PLAT002_ALERT_2_G Number of Distance or Angle Restraints on AtSite         20 Note
PLAT003_ALERT_2_G Number of Uiso or U(i,j) Restrained non-H Atoms          24 Report
PLAT007_ALERT_5_G Number of Unrefined Donor-H Atoms .............           4 Report
              H7AA  H7AB  H7A   H7B
PLAT012_ALERT_1_G N.O.K.   _shelx_res_checksum Found in CIF ......     Please Check
PLAT042_ALERT_1_G Calc. and Reported MoietyFormula Strings  Differ     Please Check
              Calc: C52 H44 Dy4 N8 O32 S4
              Rep.: C13 H11 Dy N2 O8 S
PLAT045_ALERT_1_G Calculated and Reported Z Differ by a Factor ...      0.250 Check
PLAT083_ALERT_2_G SHELXL Second Parameter in WGHT  Unusually Large     148.47 Why ?
PLAT172_ALERT_4_G The CIF-Embedded .res File Contains DFIX Records          5 Report
PLAT174_ALERT_4_G The CIF-Embedded .res File Contains FLAT Records          3 Report
PLAT176_ALERT_4_G The CIF-Embedded .res File Contains SADI Records          6 Report
PLAT178_ALERT_4_G The CIF-Embedded .res File Contains SIMU Records          2 Report
PLAT186_ALERT_4_G The CIF-Embedded .res File Contains ISOR Records          2 Report
PLAT188_ALERT_3_G A Non-default SIMU Restraint Value has been used     0.0100 Report
PLAT188_ALERT_3_G A Non-default SIMU Restraint Value has been used     0.0100 Report
PLAT299_ALERT_4_G Atom Site Occupancy Constrained at .............        0.5 Check
              S1      O1      O2      O3      O4      O5      O6      N1
              N2      C1      C2      C3      C4      C5      C6      C7
              C8      C9      C10     C11     C12     C13     H4      H5
              H7      H9      H10     H12     H13
PLAT301_ALERT_3_G Main Residue  Disorder .............(Resd    1)        92% Note
PLAT605_ALERT_4_G Largest Solvent Accessible VOID in the Structure        162 A**3
PLAT720_ALERT_4_G Number of Unusual/Non-Standard Labels ..........          2 Note
              H7AA    H7AB
PLAT721_ALERT_1_G Bond    Calc     0.86000, Rep     0.87010 Dev...       0.01 Ang.
              O7      -H7B             1_555   1_555 ........     #   38 Check
PLAT789_ALERT_4_G Atoms with Negative _atom_site_disorder_group  #         32 Check
PLAT811_ALERT_5_G No ADDSYM Analysis: Too Many Excluded Atoms ....          ! Info
PLAT822_ALERT_4_G CIF-embedded .res Contains Negative PART Numbers          3 Check
PLAT860_ALERT_3_G Number of Least-Squares Restraints .............        188 Note
PLAT869_ALERT_4_G ALERTS Related to the Use of SQUEEZE  Suppressed          ! Info
```

```
PLAT870_ALERT_4_G ALERTS Related to Twinning Effects Suppressed ..          ! Info
PLAT883_ALERT_1_G No Info/Value for _atom_sites_solution_primary .     Please Do !
PLAT941_ALERT_3_G Average HKL Measurement Multiplicity ..........        1.0 Low
```

```
   0 ALERT level A = Most likely a serious problem - resolve or explain
   1 ALERT level B = A potentially serious problem, consider carefully
   3 ALERT level C = Check. Ensure it is not caused by an omission or oversight
  28 ALERT level G = General information/check it is not something unexpected

   6 ALERT type 1 CIF construction/syntax error, inconsistent or missing data
   4 ALERT type 2 Indicator that the structure model may be wrong or deficient
   6 ALERT type 3 Indicator that the structure quality may be low
  14 ALERT type 4 Improvement, methodology, query or suggestion
   2 ALERT type 5 Informative message, check
```

It is advisable to attempt to resolve as many as possible of the alerts in all categories. Often the minor alerts point to easily fixed oversights, errors and omissions in your CIF or refinement strategy, so attention to these fine details can be worthwhile. In order to resolve some of the more serious problems it may be necessary to carry out additional measurements or structure refinements. However, the purpose of your study may justify the reported deviations and the more serious of these should normally be commented upon in the discussion or experimental section of a paper or in the "special_details" fields of the CIF. checkCIF was carefully designed to identify outliers and unusual parameters, but every test has its limitations and alerts that are not important in a particular case may appear. Conversely, the absence of alerts does not guarantee there are no aspects of the results needing attention. It is up to the individual to critically assess their own results and, if necessary, seek expert advice.

Publication of your CIF in IUCr journals

A basic structural check has been run on your CIF. These basic checks will be run on all CIFs submitted for publication in IUCr journals (*Acta Crystallographica*, *Journal of Applied Crystallography*, *Journal of Synchrotron Radiation*); however, if you intend to submit to *Acta Crystallographica Section C* or *E* or *IUCrData*, you should make sure that full publication checks are run on the final version of your CIF prior to submission.

Publication of your CIF in other journals

Please refer to the *Notes for Authors* of the relevant journal for any special instructions relating to CIF submission.

PLATON version of 15/07/2024; check.def file version of 15/07/2024

Datablock 1 - ellipsoid plot

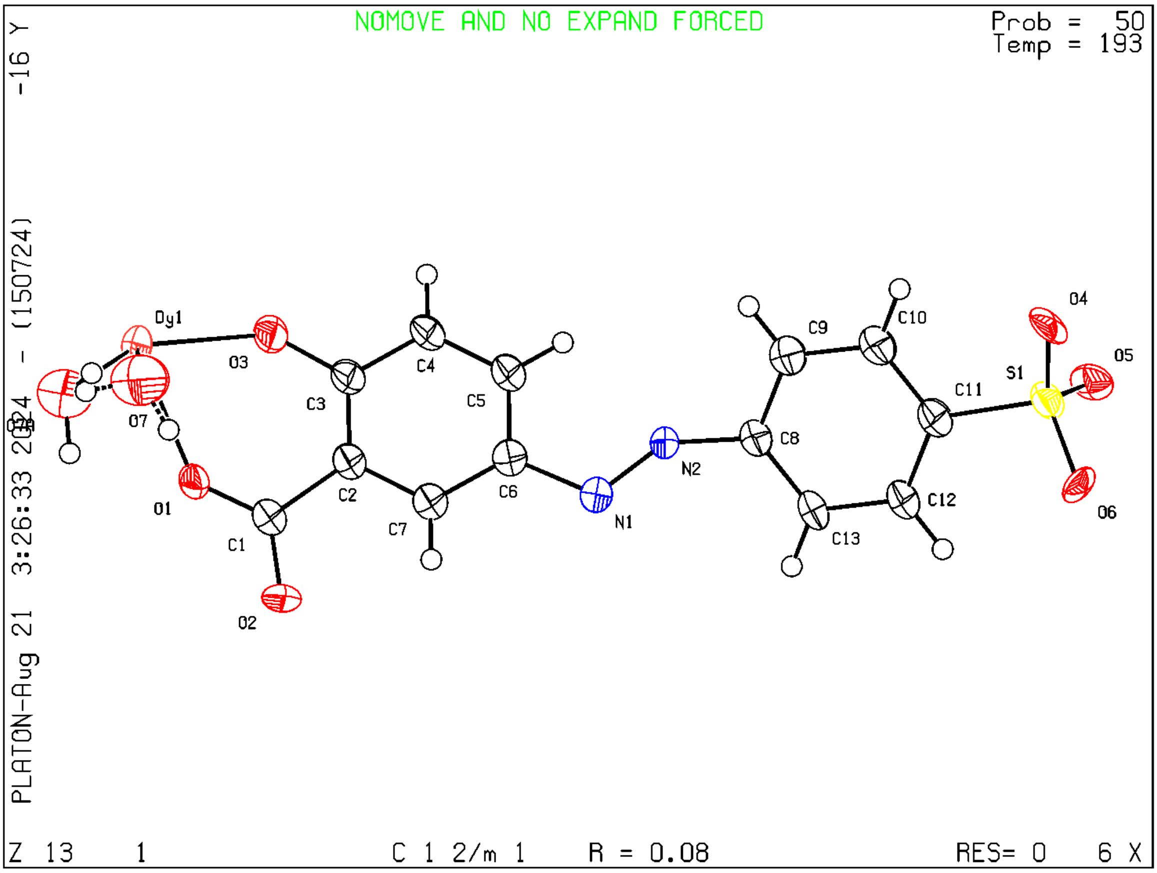

Supplement: Supplementary file 1 [file molecules-29-04495-s001.zip › checkcif of 1.pdf]
