# Supplementary material for: A Dy(III) Coordination Polymer Material as a Dual-Functional Fluorescent Sensor for the Selective Detection of Inorganic Pollutants
Source: Molecules. 2024 Sep 22;29(18):4495. doi: 10.3390/molecules29184495 (PMC11435080; doi:10.3390/molecules29184495)
Supplement: Supplementary file 1 [file molecules-29-04495-s001.zip › Supplementary Materials.pdf]

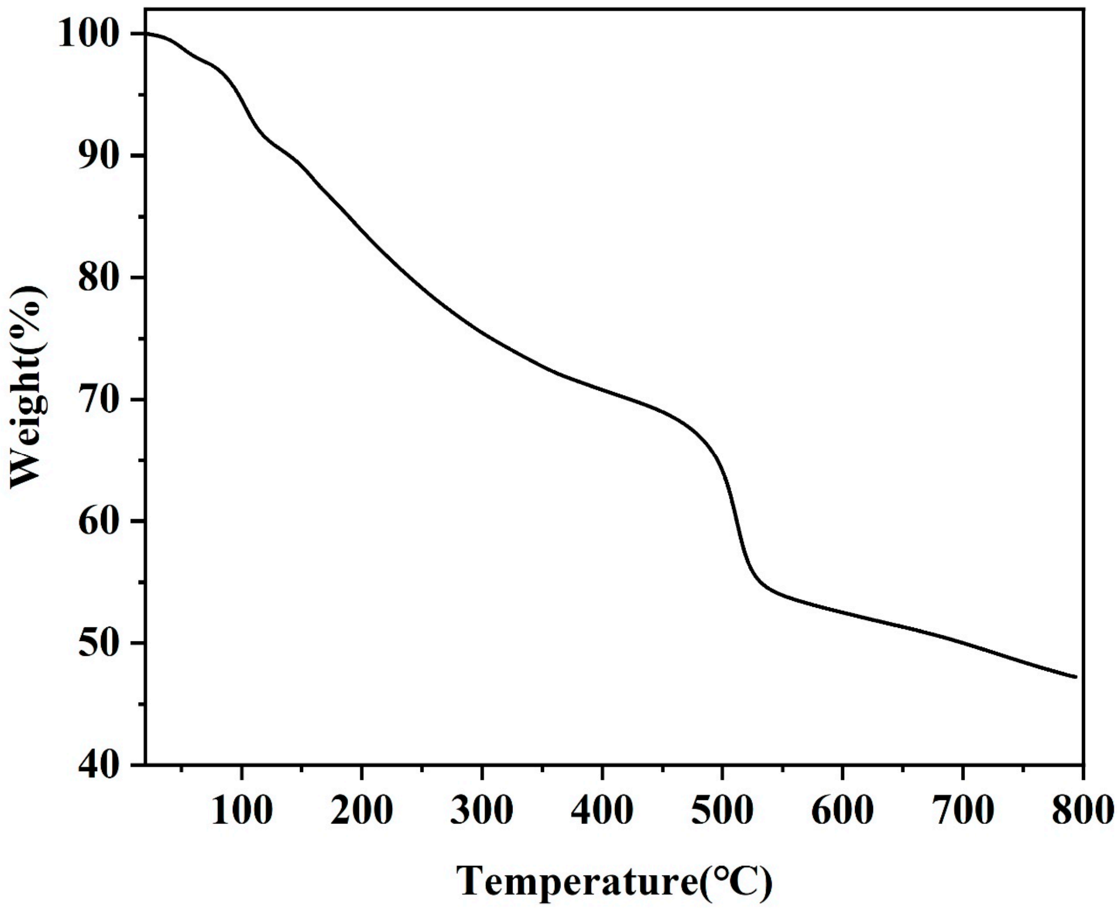

Figure S1. TGA curve of **1**.

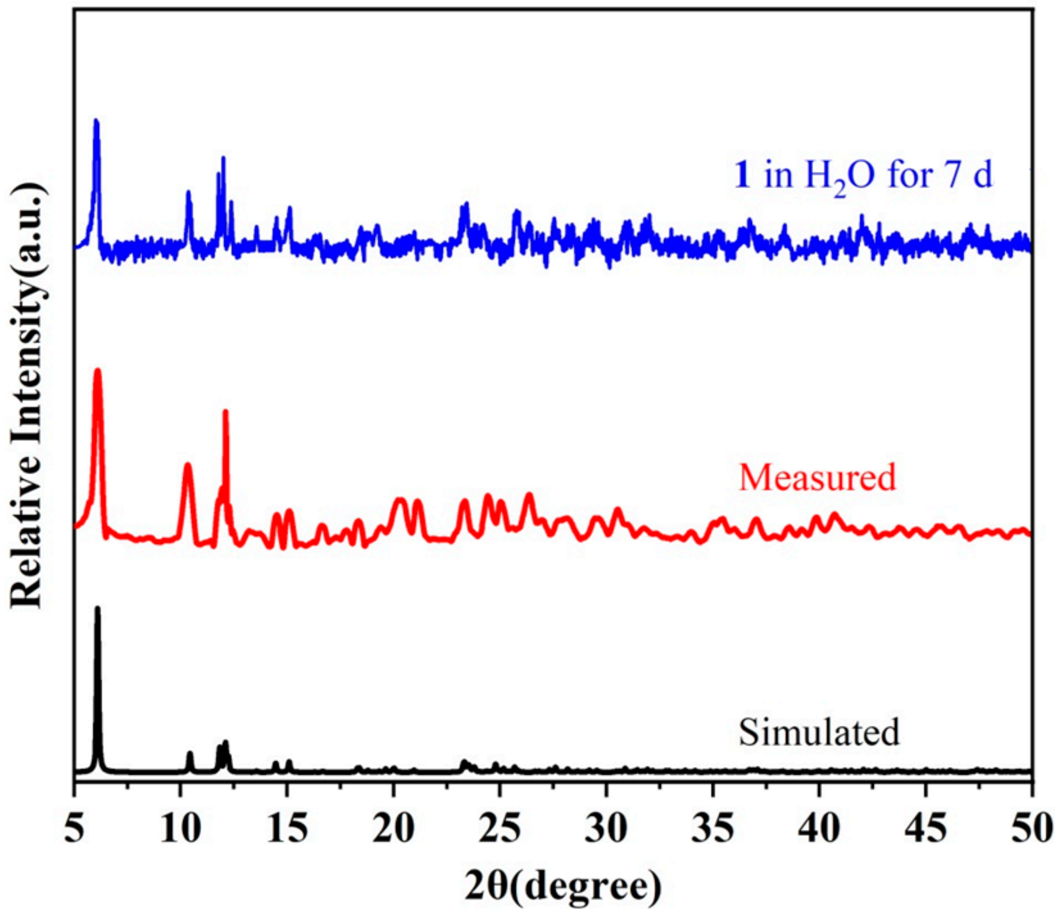

Figure S2. Measured PXRD patterns of the samples of **1** before and after soaking in water for 7 d, and simulated one based on single crystal data of **1**.

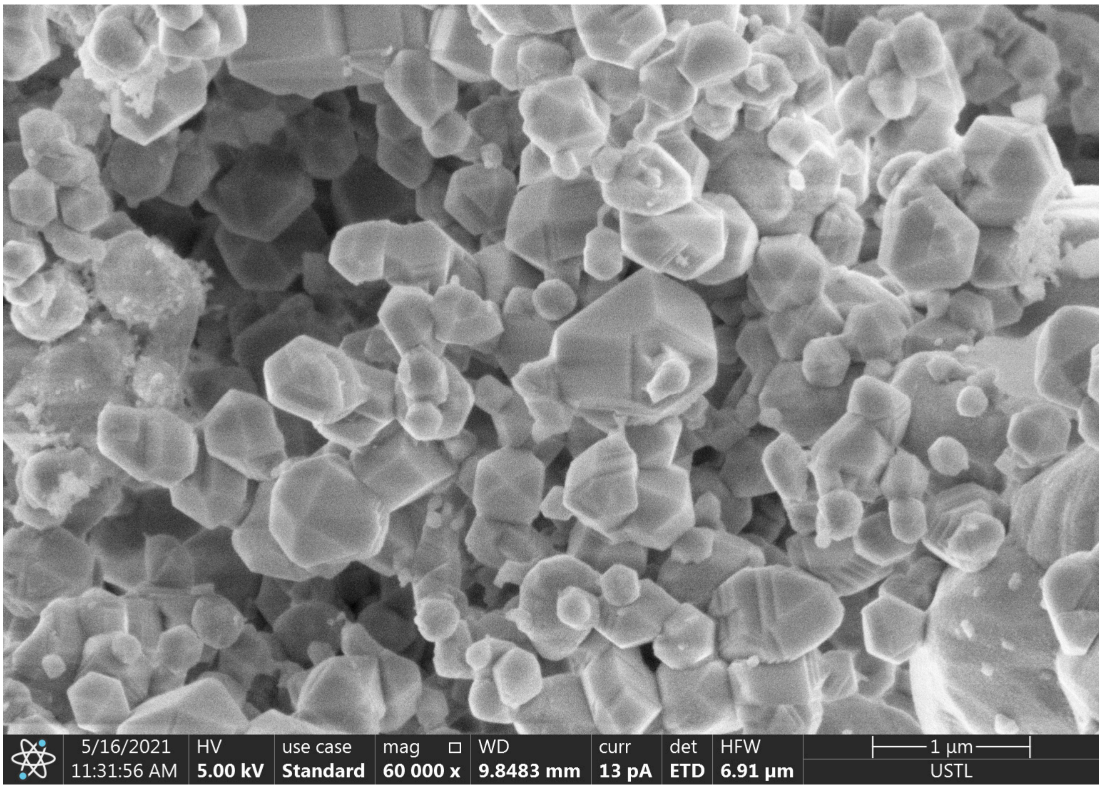

Figure S3. SEM profile of **1**.

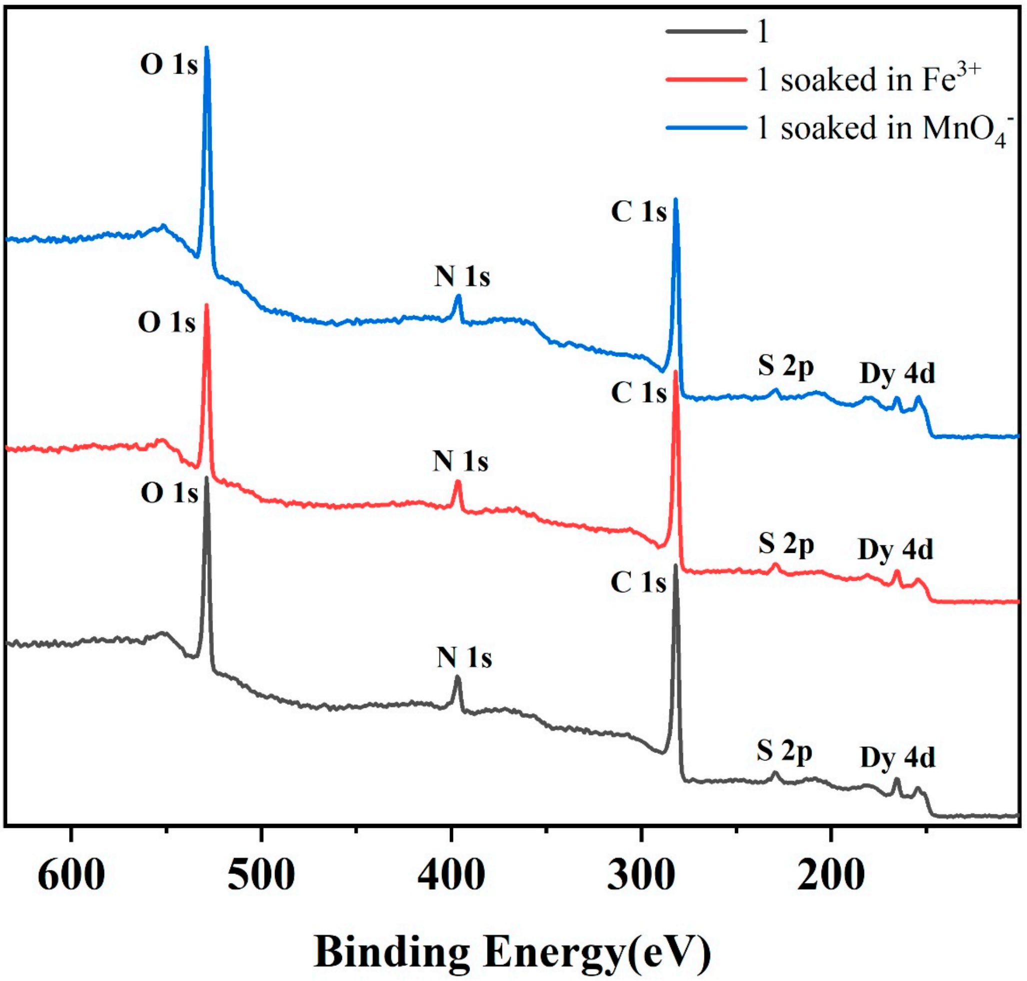

Figure S4. XPS spectra of the samples of **1** before and after treatment with Fe^{3+} and MnO_4^-.

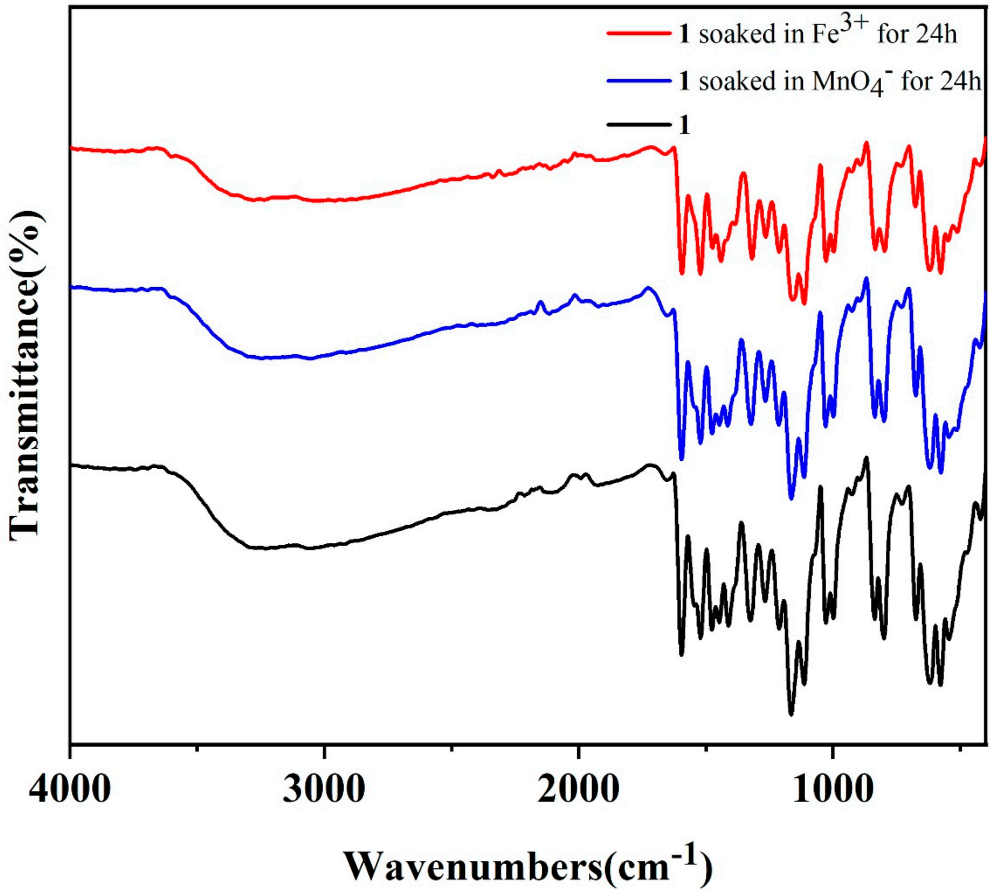

Figure S5. IR spectra of the samples of **1** before and after treatment with Fe^{3+} and MnO_4^-.

Table S1. Selected bond lengths (Å) and angles (°) for **1**.

**Atom1-Atom2**	**Distance**	**Atom1-Atom2**	**Distance**	**Atom1-Atom2**	**Distance**
Dy1-O1	2.2191(240)	Dy1-O1$^{\#1}$	2.6973(266)	Dy1-O2$^{\#1}$	2.4441(228)
Dy1-O3	2.2493(204)	Dy1-O4$^{\#2}$	2.3334(249)	Dy1-O6$^{\#3}$	2.4199(217)
Dy1-O8	2.2711(318)	Dy1-O7	2.2733(553)		
**Atom1-Atom2-Atom3**	**Angle**	**Atom1-Atom2-Atom3**	**Angle**	**Atom1-Atom2-Atom3**	**Angle**
O1-Dy1-O2$^{\#1}$	152.002(734)	O1-Dy1-O3	75.125(731)	O1-Dy1-O4$^{\#2}$	75.384(950)
O1-Dy1-O1$^{\#1}$	154.447(828)	O1-Dy1-O6$^{\#3}$	87.875(947)	O1-Dy1-O7	74.659(2044)
O1-Dy1-O8	81.516(941)	O2$^{\#1}$-Dy1-O3	132.576(747)	O2$^{\#1}$-Dy1-O4$^{\#2}$	83.849(883)
O2$^{\#1}$-Dy1-O1$^{\#1}$	50.382(647)	O2$^{\#1}$-Dy1-O6$^{\#3}$	99.596(878)	O2$^{\#1}$-Dy1-O7	92.463(2657)
O2$^{\#1}$-Dy1-O8	75.413(949)	O3-Dy1-O4$^{\#2}$	121.712(894)	O3-Dy1-O1$^{\#1}$	86.594(787)
O3-Dy1-O6$^{\#3}$	80.605(805)	O3-Dy1-O7	115.509(2012)	O3-Dy1-O8	144.339(905)
O4$^{\#2}$-Dy1-O1$^{\#1}$	130.044(853)	O4$^{\#2}$-Dy1-O6$^{\#3}$	145.909(811)	O4$^{\#2}$-Dy1-O7	103.270(1498)
O4$^{\#2}$-Dy1-O8	76.367(963)	O6$^{\#3}$-Dy1-O1$^{\#1}$	71.388(859)	O6$^{\#3}$-Dy1-O7	42.977(1251)
O6$^{\#3}$-Dy1-O8	71.865(932)	O7-Dy1-O1$^{\#1}$	98.184(2120)	O7-Dy1-O8	30.569(1521)
O8-Dy1-O1$^{\#1}$	104.981(850)				

Symmetry codes for **1**: (#1) 2−*x*, -*y*, 4−*z*. (#2) 1+*x*,1-*y*,2+*z*. (#3) 2.5−*x*,0.5 -*y*, 5−*z*.
